# How the medium shapes the message: Printing and the rise of the arts and sciences

**DOI:** 10.1371/journal.pone.0205771

**Published:** 2019-02-20

**Authors:** C. Jara-Figueroa, Amy Z. Yu, César A. Hidalgo

**Affiliations:** The MIT Media Lab, Massachusetts Institute of Technology, Cambridge, Massachusetts, United States of America; Aarhus Universitet, DENMARK

## Abstract

Communication technologies, from printing to social media, affect our historical records by changing the way ideas are spread and recorded. Yet, finding statistical evidence of this fact has been challenging. Here we combine a common causal inference technique (instrumental variable estimation) with a dataset on nearly forty thousand biographies from Wikipedia (Pantheon 2.0), to study the effect of the introduction of printing in European cities on Wikipedia’s digital biographical records. By using a city’s distance to Mainz as an instrument for the adoption of the movable type press, we show that European cities that adopted printing earlier were more likely to become the birthplace of a famous scientist or artist during the years following the invention of printing. We bring these findings to recent communication technologies by showing that the number of radios and televisions in a country correlates with the number of globally famous performing artists and sports players born in that country, even after controlling for GDP, population, and including country and year fixed effects. These findings support the hypothesis that the introduction of communication technologies can bias historical records in the direction of the content that is best suited for each technology.

## Introduction

The societal effects of communication technologies have been a matter of much discussion. Communication technologies have been argued to affect society by shaping institutions [1–5], changing the way people think [6–9], biasing the content that people transmit and record [10–13], and facilitating access to knowledge [14–19]. Yet, statistically identifying the effects of communication technologies in society has been challenging because of both, a lack of structured data on historical events and a lack of statistical instruments that can be used to address the endogeneity of the relationship between communication technology and content. Here, we use biographies of historical figures collected from online sources as a source of structured information on historical events, to study the effects of three changes in communication technologies: printing, radio, and television. The dataset we crated for this study can be accessed at *pantheon.world*.

The challenge of studying the relationship between the introduction of a communication technology, and its impact in the forms of culture produced, is one of endogeneity. Here, endogeneity refers to the challenge of knowing whether the correlation between the adoption of new communication technologies and the content produced in a given place occurs because: (i) printing causes a city to produce famous scientists and artist, (ii) because the production of famous scientists and artists causes cities to adopt printing technology, or (iii) because there are unobserved characteristics of a place that contribute to both, producing famous scientists and artists and adopting printing.

We tackle the endogeneity of the relation between communication technologies and content in the context of the invention of the movable type press by using an instrumental variable (IV), a technique that relies on an exogenous form of variation to infer the causal link between two variables. The exogenous form of variation in this case is a city’s distance to Mainz (the birthplace of printing in Europe). A priori, there is no reason to think that a city’s distance to Mainz should correlate with the probability of being the birthplace of a famous scientist or artist. In fact, prior to printing, a city’s distance to Mainz does not correlate with the probability that a city will produce a famous artist or scientists (see Section A.4 in S1 Appendix). After the invention of printing, however, the distance between a city and Mainz predicts the adoption of printing, and this predicted adoption time, subsequently, predicts the probability that a city will produce a famous scientist or artist. Of course, this does not mean that printing is the only factor that contributes to a city’s probability of producing a famous scientist or artist, but it does provide evidence in support of printing being a causal factor contributing to the probability that a city will become the birthplace of a famous scientist or artist.

For our empirical analysis, we follow Yu et al. [20] and collect data on all biographies with a presence on 15 or more Wikipedia language editions as of July 2016. The focus on historical figures as a way to measure the focus of society is justified because historical figures can be argued to embody a society’s culture and ideas [21, 22]. In particular, we collect biographical data from Wikipedia and Wikidata to study how three historical changes in media—printing (1450-1550), radio (1890-1950), and television (1950-1970)—changed the occupations of the people associated to the biographies we find in our modern digital historical records (Wikipedia and Wikidata). Only in the case of printing, however, we are able to identify the causal relationship because in that case we have an IV. What these changes in media have in common is that they had global—or at least continental—impact, and that they had relatively short adoption times [12–15, 19].

Before conducting our instrumental variable analysis we use changepoint analysis to identify discontinuities in the time series of the per-capita number of biographies associated with each year. We find that the only statistically significant discontinuity identified by this method between 500 and 1650 coincides with the introduction of Gutenberg’s movable type printing press. Then, we use distance to Mainz as an instrumental variable to measure the effect of the adoption of printing in a city’s ability to produce memorable scientists and artists. We must emphasize that there are many factors that determine the adoption of a technology such as printing, and predicting the adoption of such a technology is beyond the scope of our research. Yet, we can use the variation of the adoption of printing due to a city’s distance to Mainz to estimate the effect of printing in a city’s production of famous scientists and artists [23]. While a city’s ability to produce famous scientists and artists may depend on a large number of variables, such as its population and wealth, that city’s distance to Mainz can only affect the number of famous people born in that city through the adoption of printing: a necessary condition for an instrumental variable, often referred to as the *exclusion restriction* [23]. While it is rarely possible to prove the exclusion restriction, the fact that the correlation between a city’s distance to Mainz and the number of scientists and artists born in that city only appears after the invention of printing, helps rule out other mechanisms, such as the distance to Mainz correlating with the presence of universities that provide infrastructure for the arts and sciences. Hence, a city’s distance to Mainz is an exogenous form of variation that increases the likelihood of being an early adopter of printing technology. In fact, between 1450 and 1500, printing diffused in concentric circles as printers set out to establish presses in other cities [18]. Therefore, it is not surprising that distance to Mainz has been used to establish the impact of the printing press in economic growth [18], and on the spread of Protestantism [24]. Our two stage least square estimate using distance to Mainz as an instrumental variable confirms the hypothesis that cities that adopted printing earlier produced famous scientists and famous artists earlier, indicating that printing promoted the emergence of a scientific and artistic elite.

To bring these findings to other eras we explore the occupations associated to biographies from each communication era (writing, printing, radio, and television), finding that each era is characterized by its own composition of famous occupations. Finally, we use data at the country level on the number of radios and the number of televisions available each year in each country [25] to show that countries with more radios and with more televisions, were the birthplace of more famous artists and sports players. The correlation remains strong even after controlling for GDP, population, and including country and year fixed effects, which control for unobserved time-invariant country characteristics and for unobserved time-varying world characteristics respectively. The same is not true for occupations such as scientists, that experienced an increase with the rise of printing, but not with radio or television. While we are not able to identify the causal effect for radio and television, we show correlational evidence that complements the causal identification for the effect of printing. These results support the idea that communication technologies shape our historical records by biasing their content towards the type of information best suited for the media of each time.

## Data

We use biographical data from two different datasets: the *Pantheon 2.0* dataset, which we have collected for this project, and the *Human Accomplishments* dataset [26]. The *Human Accomplishments* dataset [26] contains more than three thousand biographies of individuals from the Arts and Sciences that are recorded in authoritative printed texts in six different languages. The Pantheon 2.0 is an extension of the Pantheon 1.0 dataset [20]. Pantheon 1.0 is a peer-reviewed dataset containing the 11,337 biographies that had a presence in more than 25 different language editions of Wikipedia as of May 2013 (for more details see [20]). Pantheon 2.0 extends the 1.0 version to include people with 15 language editions or more in Wikipedia as of July 2016. As in Pantheon 1.0, a biography is any Wikipedia page about a single individual. The Wikipedia page about the “Wright brothers,” for example, is not considered a biography because it is not about a single person. The Pantheon 2.0 dataset associates each biography with a city of birth, a date of birth, and an occupation. The place and date of birth are taken from the information provided in each biography’s Wikipedia infobox, complemented with the information present in each biography’s Wikidata page. The city of birth is obtained by associating each place of birth to a city from the GeoNames database [27] (we use cities over 5000 people). We assign occupations using a machine learning classifier that extracts features from the biography’s text on Wikipedia. We trained the classifier on the Pantheon 1.0 dataset. By using information from the entire Wikipedia page of a person’s biography, our algorithm captures how this person is remembered by the people who have edited this biography. For example, Pablo Neruda is described as both a diplomat and a poet in his Wikipedia page, but most of the text is about his career as a writer and not as a diplomat. Therefore, our algorithm correctly detects the focus of the biography and assigns Neruda to the “writer” occupation. For our analysis, we aggregate occupations into 8 categories: political leaders, religious figures, scientists, performing artists, fine artist, humanities, and sports. We must note that classical composers such as Beethoven and Mozart are included under the fine artists category. More details about the data collection for Pantheon 2.0 can be found in Section A.1 in S1 Appendix.

Both of these datasets add to a recent stream of literature focused on the use and development of quantitative methods to explore historical patterns and the success of creative efforts [20, 26, 28–35]. Examples of these studies include the evolution of language and ideas as recorded in printed books [29], patterns of historical migration [32], the importance of language translations in the global diffusion of information [36], the emotional content of global languages [37], and the dynamics of fame [20, 28, 38]. These studies are made possible thanks to new digital sources such as Wikipedia, Freebase, Wikidata, and digitized books [39].

Data on technology adoption comes from two sources. For printing, we use the *Incunabula Short Title Catalogue* [40], a dataset comprising all books printed between 1450 and 1500. This data is suitable to understand the adoption of printing because the period 1450–1500 (the “first infancy” of printing [41]) was a period of rapid expansion of printing among European cities, with the price of books falling by nearly two thirds [42, 43]. For radio and for television we use the Historical Cross-Country Technology Adoption (HCCTA) dataset [25], a dataset collected to analyze the adoption patterns of some of the major technologies introduced in the past 250 years. The HCCTA dataset also has historical GDP and population information.

Population data comes from two sources. At the global level we use data from the historical world population estimates of the US Census Bureau [44], which reports an aggregated dataset of world population estimates starting from the year 10,000 BC. At the city level we use a dataset on population of urban settlements from 3700 BC to AD 2000 [45]. We matched the cities present in [45] with the ones in [27] based on their name and coordinates.

All of our data sets have limitations that need to be considered when interpreting our results. Therefore, we emphasize the need to interpret our results as valid only in the narrow context of the sources used to compile these datasets. The Pantheon 2.0 dataset has all the biases inherent in using Wikipedia as our main data source [20], therefore our results should be interpreted as a reflection of the historical records that are representative only of the people who edit the more than 250 language editions of Wikipedia—a literate and digitally empowered elite of knowledge enthusiasts [46]. Moreover, Wikipedia is not a primary data source for historical research. Nevertheless, Wikipedia editors have developed a complex set of rules and criteria to decide what information gets recorded [47, 48], and thus Wikipedia data can be used to map the focus of society [49, 50]. The Human Accomplishments dataset is compiled from printed encyclopedias in six different languages, and is representative of the people who participated in the creation of printed encyclopedias. An extended discussion of the biases and validation of the Human Accomplishments dataset can be found in [26].

## Results

We study the global effects of printing by looking at the number of biographies *B* born in a given time window Δ*T* for both the Pantheon 2.0 and the Human Accomplishment datasets. To make these estimates comparable across time we normalize *B* by the average world population *N* and the size of its respective time window. Hence, we define $m=\frac{B}{N\Delta T}$. *m* is an estimate of the per-capita number of births occurring in a given year that resulted in a biography that is prominently recorded in each of our datasets. *m* is measured in units of births per year per billion people in the world, or [bpyb].

**Fig 1:**
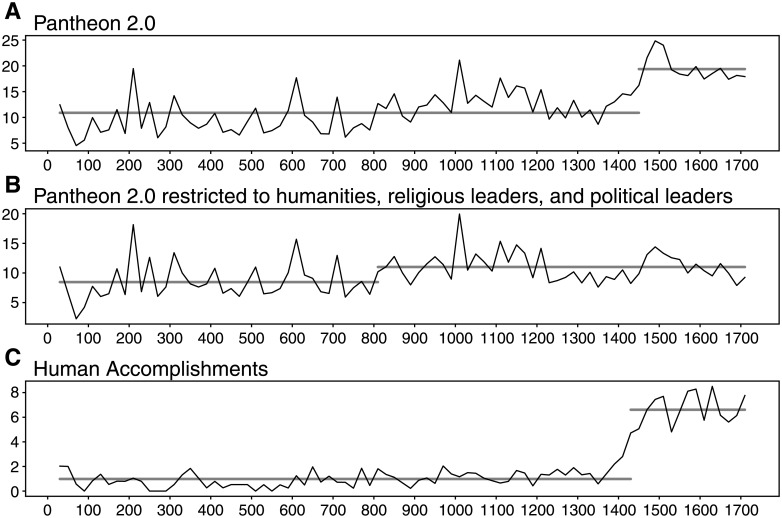
Per-capita births of globally memorable people. The per-capita births of globally memorable people (*m*) is measured using a 20-years time window for (A) the full Pantheon 2.0 dataset, (B) Pantheon 2.0 dataset restricted to **government**, **humanities**, and **religion**, and (C) the Human Accomplishments dataset. The horizontal lines correspond to the average for each part of the time series, as detected by the changepoint analysis.

Fig 1A and 1C show the temporal evolution of *m* as observed in both datasets between year 1 and 1730, using a 20-years time window. In both datasets *m* is constant for the 1,500 years preceding the introduction of the movable type press but increases by more than 50% together with the introduction of printing according to our changepoint analysis [51]. Furthermore, we find that once we limit the Pantheon 2.0 dataset to only political and religious leaders–the two categories that were prominently present in the dataset prior to printing (see Fig 3A)–there is no statistically significant break (see Fig 1B). This suggests that the increase in the number of per-capita biographies observed with the rise of printing was the result of a new pattern in reading habits that was genre specific.

**Fig 2:**
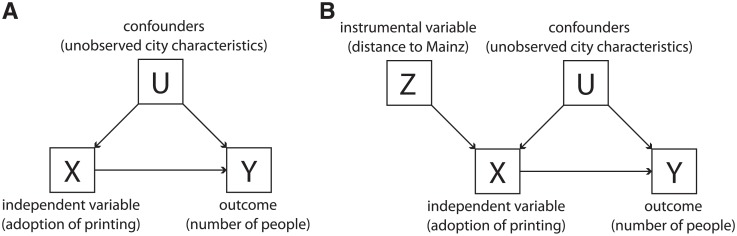
Directed acyclic graph (DAG) diagram of our instrumental variable strategy. (A) shows the potential for confounding factors arising from unobserved city characteristics that correlate with the adoption of printing and with the number of memorable characters born in each city. In this situation, a simple regression of *Y* on *X* would not yield the correct estimation of the causal effect of *X* on *Y* because of the “backdoor path” introduced by *U*. (B) shows the introduction of an instrumental variable *Z* that correlates with *X*, but does not correlate with *Y* directly nor through *U*.

Next we address endogeneity concerns by using the distance to Mainz as an instrumental variable [18, 23, 24, 52]. The need for such an approach is highlighted in Fig 2A and comes from potential unobserved city characteristics that can correlate with both: the likelihood of adopting printing and the number of memorable characters of each occupation born in each city. For example, the wealth of a city can lead both to the city being an early adopter of printing and the city being the birthplace of many scientists. When introducing a city’s distance to Mainz as an instrument for the adoption of printing (Fig 2B), we use the correlation between the adoption of printing and a city’s distance to Mainz as a first stage to estimate the effect of printing in a city’s ability to produce memorable characters in different occupations. In other words, we first regress the adoption of printing on the distance to Mainz, and then use the predicted values for the adoption of printing as independent variables in a regression with the number of memorable characters as outcome variable. This approach correctly identifies the causal effect without the need of controlling for confounders [23, 52]. In practice, we use two different outcome variables calculated at the city level to quantify early adoption of printing (i.e., as treatment variables): a dummy variable for adopting printing between 1450 and 1500, and the year of the first printed book (for a third empirical specification see Section B.1 in S1 Appendix).

Our first instrumental variable analysis uses a city’s distance to Mainz as an instrument for adopting printing between 1450 and 1500. Data on printing adoption is obtained from the Incunabula Short Title Catalogue [40]. As dependent variables we use the number of scientists, artists, and political leaders born in each city between 1450 and 1550, right after the invention of printing (for the other occupations see Section B.1 in S1 Appendix). If the introduction of printing increased the probability that a city would become the birthplace of a famous scientist or artist, then we should observe a significant effect in the number of scientists and artists, but not in the number of political leaders born in a city. Political leaders were already prominent prior to printing. Table 1 shows the first stage, and the OLS and IV estimators for each dependent variable. We see that adopting printing earlier (before 1500) has a significant, positive, and strong effect on the number of scientists and artists, but does not have a significant effect on the number of political leaders born in a city, meaning that early adopters of printing were more likely to become the birthplace of famous scientists and artists than late adopters of printing.

**Table 1:** Instrumental variable analysis of the effect of printing on European cities. Here, we use the distance to Mainz as an instrument for a dummy variable for adopting printing between 1450 and 1500. We use a two stage least squared regression to estimate the effect of adopting printing between 1450 and 1500 on the number of scientists, artists, and political leaders born in each city between 1400 and 1550. All dependent variables are in logarithmic scale.

	*Dependent variable*:							
	printing before 1500		number of scientists		number of artists		number of political leaders	
	*probit* (1)	*First Stage* (2)	*OLS* (3)	*IV* (4)	*OLS* (5)	*IV* (6)	*OLS* (7)	*IV* (8)
distance to Mainz	−0.273*** (0.033)	−0.022*** (0.003)						
printing before 1500			0.089*** (0.004)	0.222*** (0.043)	0.202*** (0.007)	0.302*** (0.067)	0.188*** (0.009)	0.130 (0.081)
constant	−0.014 (0.212)	0.186*** (0.018)	0.002* (0.001)	−0.003 (0.002)	0.003* (0.001)	−0.0002 (0.003)	0.010*** (0.002)	0.012*** (0.003)
Observations	5,335	5,335	5,335	5,335	5,335	5,335	5,335	5,335
Log Likelihood	−804.19							
AIC	1,612							
Residual SE		0.187	0.061	0.066	0.100	0.102	0.124	0.124
R^2^		0.012	0.070		0.125		0.075	
Adjusted R^2^		0.012	0.070		0.125		0.075	
F Statistic		66.57***	399.43***		762.57***		435.08***	

Note:

*p<0.05;

**p<0.01;

***p<0.001

For our second instrumental variable analysis we use distance to Mainz as an instrument for the year of the first printed book. This analysis is restricted to cities that adopted printing, so the number of observations decreases considerably. As dependent variables we use the year of the first recorded scientist born in each city, the year of the first recorded artist born in each city, and the year of the first political leader born in each city (for the other occupations see Section B.1 in S1 Appendix). Adding to our previous results, Table 2 shows that the year of the first printed book has a significant, positive, and strong effect on the birth of famous scientists and artists, but does not have a significant effect on the birth of famous political leaders.

**Table 2:** Instrumental variable analysis of the effect of printing on European cities that adopted printing. Here, we use the distance to Mainz as an instrument for the year of the first printing press in each city, and use it to estimate the effect of printing in the birth of scientists, artists, and political leaders.

	*Dependent variable*:						
	year of first printer	year of first scientist		year of first artist		year of first political leader	
	*First Stage* (1)	*OLS* (2)	*IV* (3)	*OLS* (4)	*IV* (5)	*OLS* (6)	*IV* (7)
distance to Mainz	0.010** (0.003)						
year of first printer		4.870** (1.425)	12.900* (5.655)	6.552*** (1.740)	12.956* (6.194)	3.036 (1.809)	−1.955 (4.988)
population	−3.280* (1.644)	−18.507 (19.035)	−5.089 (24.651)	−37.476 (21.950)	−38.083 (24.072)	−64.311* (26.015)	−68.651* (27.613)
constant	55.853*** (16.207)	294.220 (203.821)	−69.230 (346.180)	383.895 (231.540)	215.241 (297.534)	719.812* (275.779)	908.366** (337.974)
Observations	82	73	73	70	70	77	77
R^2^	0.132	0.163		0.202		0.113	
Adjusted R^2^	0.111	0.139		0.178		0.089	
F Statistic	6.032**	6.833**		8.496***		4.732*	
F Statistic df	2; 79	2; 70		2; 67		2; 74	

Note:

*p<0.05;

**p<0.01;

***p<0.001

**Fig 3:**
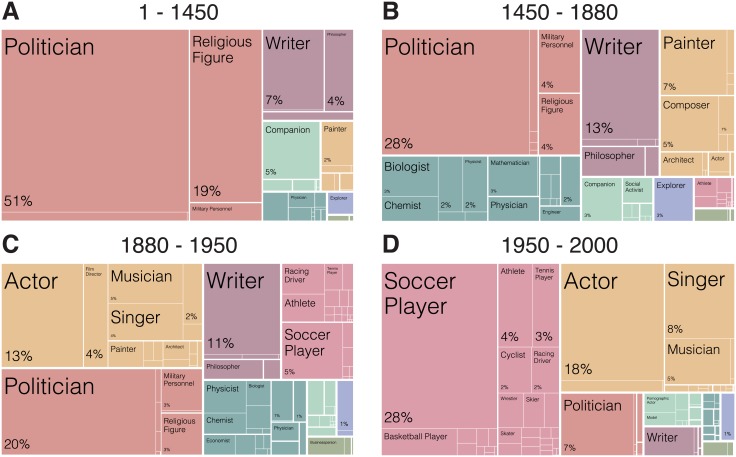
Composition of occupations for the biographies in Pantheon 2.0. For the period between (A) 1-1450, (B) 1450-1880, (C) 1880-1950, and (D) 1950-2000.

Next, we look at the occupations associated with the biographies in Pantheon 2.0 dataset at each period in time. Fig 3 shows the fraction of biographies corresponding to each occupation category for each period split into the technological eras defined by the introduction of communication technologies. First, comparing Fig 3A and 3B we see that printing is associated with an increase in the fraction of painters, composers, and scientists (such as physicists, mathematicians, and astronomers), in Wikipedia’s biographical records. It is also, associated with a decrease in the fraction of religious figures. Second, radio, a technology spread at about the same time as film, (Fig 3B and 3C) was accompanied by a shift towards the performing arts and an increase in the fraction of actors, singers, and musicians in Wikipedia’s biographical records. Finally, with the introduction of television (Fig 3C and 3D) we see the rise of sports players—such as soccer players, basketball players, and race-car drivers—and the consolidation of performing artists.

Table 3 shows the results of regressing the number of performing artists born each year in each country, the number of sports players, and the number of scientists, on the number of radios, and the number of televisions. Since we are dealing with panel data, all models in Table 3 include country fixed effects and year fixed effects. Country fixed effects control for unobserved time-invariant country characteristics, and year fixed effects control for time-varying world characteristics. The results show that, after controlling for GDP and population, countries that had more radios and television were the birthplace of more famous performing artists, and sports players. Moreover, radios and televisions have no correlation with the number of scientists born each year in each country. In fact, the difference in the explanatory between models (8) and (9) from Table 3 is not significant (p-value∼0.19), while the difference between models (2) and (3), and (5) and (6) are significant (p-value∼10^−16^). Despite the fact we have added two time-varying country level controls (population and GDP), there may be other unobserved confounding factors that may render our correlations spurious. Nevertheless, our results provide evidence of the link between radio and performing arts, and between television and sports, which could be the matter of future studies.

**Table 3:** Correlation between number of radios and television and the birth of memorable characters. We explore the correlation between the number of radios and televisions a country has each year, with the number of performing artists, sports players, and scientists born each year, after controlling for GDP, population, and adding country fixed effects and year fixed effects. Reported errors correspond to robust standard errors. All quantities are calculated yearly between 1850 and 1973, and are in log scale.

	*Dependent variable*:								
	number of performing artists			number of sports players			number of scientists		
	(1)	(2)	(3)	(4)	(5)	(6)	(7)	(8)	(9)
n. of radios	0.037*** (0.005)		0.031*** (0.005)	0.027*** (0.004)		0.029*** (0.004)	0.003 (0.004)		−0.003 (0.004)
n. of televisions	0.079*** (0.009)		0.072*** (0.009)	0.040*** (0.006)		0.042*** (0.006)	−0.001 (0.006)		−0.007 (0.006)
GDP		0.477*** (0.069)	0.312*** (0.067)		0.091 (0.050)	−0.033 (0.050)		0.218*** (0.043)	0.235*** (0.044)
population		−0.283*** (0.070)	−0.158* (0.068)		−0.135* (0.053)	−0.041 (0.052)		−0.044 (0.044)	−0.056 (0.044)
constant	−0.144 (0.099)	0.154 (0.573)	−0.310 (0.538)	−0.198*** (0.052)	0.965* (0.377)	0.599 (0.366)	−0.017 (0.101)	−1.046** (0.362)	−1.001** (0.364)
fixed effects:									
country	✓	✓	✓	✓	✓	✓	✓	✓	✓
year	✓	✓	✓	✓	✓	✓	✓	✓	✓
Obs.	3,011	3,011	3,011	3,011	3,011	3,011	3,011	3,011	3,011
R^2^	0.638	0.622	0.645	0.576	0.551	0.578	0.539	0.556	0.557
Adj. R^2^	0.615	0.599	0.623	0.549	0.523	0.552	0.511	0.529	0.529
F Statistic	28.49***	26.65***	29.10***	21.97***	19.90***	21.92***	18.98***	20.29***	20.09***
F Statistic df	175	175	177	175	175	177	175	175	177

Note:

*p<0.05;

**p<0.01;

***p<0.001

## Discussion

The famous Canadian philosopher of communication Marshall McLuhan coined the phrase “the medium is the message” to convey his belief that communication technologies were more consequential to society than the messages uttered through them. In this paper we follow this tradition by statistically studying how communication technologies affect the biographies present in our modern digital historical records. As noted earlier, we are by no means the first ones to tackle this question [6–19, 21, 31, 53–57]. Nevertheless, we contribute to this literature by using two large biographical datasets (one of them for the first time), together with an instrumental variable approach to quantify the effect of the introduction of printing, radio, and television in the volume and composition of the biographies present in modern digital historical records.

As previously noted by Lance Strate [21, 57], communication technologies change the characteristics of our “heroes.” From oral culture to broadcasting, the “hero” is a human figure that serves as an object of admiration, aspiration, and at times, worship [21, 22, 57]. Strate notes that because oral culture was concerned with the preservation of information, oral heroes are “heavy” figures; memorable, larger than life, mythological, and characterized by their incredible actions. Most of them are either kings in charge of ruling vast empires, or religious figures that have performed impossible deeds (see Fig 3A). With the introduction of the printing press literacy replaced orality and heavy figures were no longer necessary. Literacy allowed for a greater number of heroes (see Fig 1), each of whom were more realistic and sensible. The tales of kings and saints became enriched with stories of ordinary people pursuing a larger diversity of careers [15] (see Fig 3B). During this time, ideas became more important than actions, giving way to printing heroes such as the author, inventor, composer, and scientist [22].

The spread of printing was fast across Europe. In less than forty years the price of books fell by two-thirds, transforming the ways in which ideas and data were disseminated, and thus the conditions for intellectual work. The social and economic effects of printing were discussed in detail by the printing historian Elizabeth Eisenstein [14, 15] who argued that printing not only changed the number of books printed during the Renaissance, but also who the authors of these books were and what these books were about. According to Eisenstein, printing shifted the relative focus of society away from religious texts—which were a staple output of the scribal culture that preceded printing—and towards more art and science books. Printing scaled up the production of all texts, including religious texts, but the access to secular books grew at a much higher rate than the access to religious books.

Printing not only provided a new complementary media for scientists and artists to better disseminate their work, but it also enabled the proliferation of academic resources. These two mechanisms contributed to making cities that were early adopters of printing the birthplaces of scientists and artists (see Table 2). Our results show precisely that. Moreover, we find that printing had no effect in a city’s chance of being the birthplace of a memorable political or religious leader (see Tables 1 and 2, for religious leaders see Section B.1 in S1 Appendix).

Printing is often argued to be one of the most “access cost-reducing inventions” in history [16]. Because of this, historians have argued that printing boosted the sciences and arts by facilitating a “combinatory intellectual activity” [14–16, 58]. In fact, the same specification strategy used in this paper was used to show that early adoption of printing led to higher GDP growth rates [18]. In economic terms, cities that adopted printing earlier benefited from localized spillovers in human capital accumulation, leading to a higher chance of producing a successful scientist or artist. Despite the impact of printing being a generally accepted fact, it would be unfair to say that printing created the modern world [16]. Our work contributes to this debate by showing that printing was indeed one factor, without claiming it was the only one.

The second and third changes in media studied here were the introduction of radio and film in the early twentieth century, and the introduction of television in the second half of the twentieth. Film gave rise to the star system, when after 1910 certain identifiable movie actors began to be preferred over others [55]. Radio and television, on the other hand, favor fast dissemination of information over space, rather than preservation over time [13, 21]. When content is quickly flashing in and out of attention, there is little time to focus on ideas. Instead, images and performances become more relevant. This is something we observe in our data, as society moves to broadcasting technologies, performers take a bigger portion of history (see Fig 3C).

Television has been argued to strongly favor entertainment content [12, 53, 54]. Neil Postman discussed the societal effect of television in the United States, and argued that television not only created new types of content, such as sports, but also affected existing types of content. News reporting on television, for example, favored simple sentences to make reading on the air more effective [54], as opposed to the more complex sentences that were common in newspapers. Because television favored entertainment, it should be associated with the biographies of performers, such as actors and singers, and with biographies of sports players, but not with the biographies of scientists, whose content is not as fitting for television as that of entertainers. Our results show that even after controlling for population and GDP, the number of televisions and radios present in a country correlate with the number of performers and sports players born in each country. Moreover, the correlation between television and sports players is stronger than between radios and sports players (see Table 3). In some sense, television “invented” sports stars, since due to its immediate nature [12] it favored entertainment content that was more valuable when consumed live.

We have discussed the impact of three major changes in media: printing press, film and radio, and television. Something these changes in media have in common is that the changes in the content that was recorded following each one always favored the occupations of those who can best express their work using the new media. The rise of printing, for instance, was followed by more composers recorded in our historical records, but not by more singers or musicians (see Fig 3). In the same way, film promoted the actor over the play-writer. In fact, changes in media helped include in our historical records occupations that had long existed, but that were not prominently recorded. For example, both Ancient Greek and Elizabethan English playwrights employed actors, but performers were not recorded in the absence of a technology capable of recording performances—such as film.

Our findings also contribute to the discussion around the idea of technological determinism [59]. Technological determinism is the problem of identifying causal relations between technology and society [60], and is marked by a tension between two opposing views [59]. On the “hard” end of the spectrum, we have the idea that the power to affect change is in the technology itself. On the “soft” end, the agency is not in the technology, but in a far more complex array of social, political, economical, and cultural factors. Under this soft view, technology is as a mediating factor, rather than the driving force since it mostly reflects the influence of socioeconomic forces that drive history [61]. Our work clearly identifies a causal relation between the spread of printing and the occupations of people that are recorded by history, thus documenting a clear societal impact of a new technology. This impact is not economic or political, but cultural, and can thus add to the debate about technological determinism.

Finally, we note that observations for most recent years need to be interpreted carefully for two reasons. First, the more recent biographies in our dataset contain a mix of characters that are memorable (e.g. Barack Obama, as the first African American president of the United States) with characters whose presence in today’s biographical records may not necessarily be long lasting (like teen pop icons and reality show celebrities). So the picture obtained for recent decades is not the one we expect to be representative of those decades in the future. Nevertheless, we can safely assume that this issue does not affect our historical data prior to the twentieth century, since these transient effects should not last for centuries after a person’s death. Second, we note that data for the most recent years is also affected by differences in the life cycle of an individual’s memorability, since individuals with different careers peak at different ages. Soccer players, for example, peak around their late twenties or early thirties [62], so our dataset should contain all soccer players born in the 1950s that became memorable players. Political leaders and scientists, on the other hand, often become globally memorable much later in life [63], and hence, we may be missing some influential individuals who are yet to reach global recognition. Both of these effects imply that fifty years from now the fraction of our biographical records allocated to sports players will be smaller than what we observe in our data today. In other words, we expect the biographical records to change as time continues to elapse.

Prior to printing, our heroes were part of the most powerful institutional elites in the world. Now, we live in a world in which history is almost personalized, since billions of individuals now leave traces that could be used to reconstruct biographical data through personal acts of communication (emails, text messages, and social media posts). Of course, this does not mean that everyone will become memorable, but maybe that memorability will now have a chance to spread over a wider number of people who may now enjoy intermediate levels of memorability and fame. This is an effect that has already been observed in the context of creative industries [64]. Going forward, the rise of digitized historical records will help us continue the statistical study of these and other hypotheses, and maybe guide our speculation about the nature of the heroes of the future.

## Supporting information

S1 Appendix Online Appendix includes: 1) Extended data and methods, 2) Supplementary results, and 3) Table of occupations for Pantheon 2.0. (PDF)
