# Supplementary material for: How the medium shapes the message: Printing and the rise of the arts and sciences
Source: PLoS One. 2019 Feb 20;14(2):e0205771. doi: 10.1371/journal.pone.0205771 (PMC6382096; doi:10.1371/journal.pone.0205771)
Supplement: S1 Appendix — (PDF) [file pone.0205771.s001.pdf]

How the medium shapes the message: Printing and the rise of the arts and sciences
Online Appendix

C. Jara-Figueroa[1], Amy Z. Yu[1], and César A. Hidalgo[1,*]

[1]The MIT Media Lab, Massachusetts Institute of Technology, Cambridge, MA 02139
[*]Corresponding author: hidalgo@mit.edu

November 5, 2018

Abstract

Communication technologies, from printing to social media, affect our historical records by changing the way ideas are spread and recorded. Yet, finding statistical evidence of this fact has been challenging. Here we combine a common causal inference technique (instrumental variable estimation) with a dataset on nearly forty thousand biographies from Wikipedia (Pantheon 2.0), to study the effect of the introduction of printing in European cities on Wikipedia's digital biographical records. By using a city's distance to Mainz as an instrument for the adoption of the movable type press, we show that European cities that adopted printing earlier were more likely to become the birthplace of a famous scientist or artist during the years following the invention of printing. We bring these findings to recent communication technologies by showing that the number of radios and televisions in a country correlates with the number of globally famous performing artists and sports players born in that country, even after controlling for GDP, population, and including country and year fixed effects. These findings support the hypothesis that the introduction of communication technologies can bias historical records in the direction of the content that is best suited for each technology.

Contents

A Extended Data and Methods **2**
- A.1 Pantheon . . . 2
- A.2 Population and technology adoption . . . 3
- A.3 Changepoint analysis . . . 3
- A.4 Distance to Mainz as an IV . . . 3

B Supplementary results **6**
- B.1 Printing and the rise of the arts and sciences . . . 6
- B.2 Radio and television and the rise of entertainment . . . 6

C Table of occupations **12**

A Extended Data and Methods

A.1 Pantheon

The Pantheon 2.0 dataset contains all the biographies that had a presence in more than 15 language editions of Wikipedia as of July 2016. As a starting point, we collected all the articles that belong to the WikiProject Biography from the English Wikipedia, and complemented this with all the *instances of* of the class *human* from Wikidata. Next, we use the Wikipedia API to collect the number of language editions for all biographies, and select only those biographies present in more than 14 language editions.

The articles within the scope of the WikiProject Biography, however, are not guaranteed to be about individual persons. For example, articles about music bands or duos, lists of monarchs, or terrorist groups are sometimes within the scope of the WikiProject Biography. To filter out the articles that do not correspond to a biography of a single person we manually check all the articles for which: i) we cannot find a *gender*, ii) the title that starts with the word "list," or that includes either "and" or "&", iii) contain either the word "band," "duo," or "group" after the verb "to be" in the first sentence of the description, and iv) the verb "to be" from the first sentence appears in its plural from.

Next, we assigned each biography to their year of birth and their place of birth using the Wikipedia Infobox present in most of the biographies. When the infobox was no available we used Wikidata. The granularity of the place of birth varies across briographies, with some biographies been assigned to countries, others to cities, and others to precise locations with cities (for example, a palace within a city). To use a controlled set for the cities, we associate each place of birth to a city from the GeoNames database (1). In particular, we use *cities over 20000 people* (defined in GeoNames as settlements with a present day population of more than 20000 people). The assignment is done by matching the name of the place of birth with the name of the city, within a 30km radius. When this method failed, the place of birth was manually assigned. Countries, regions, and continents were not assigned to any city.

Finally, we describe the method used to obtain the occupations associated to the biographies in Pantheon 2.0. The occupations associated with characters in Pantheon are meant to capture the way the character is recorded in our historical records. Many characters hold an occupation that is not what they are remembered for. For example, Margaret Thatcher is a chemist and a lawyer, but she is recorded in our collective memory as the longest-serving British Prime Minister of the 20th century, and the only woman to have held that office so far; she is recorded as a politician. The available databases that contain information about historical characters, such as Freebase, Wikidata, and DBpedia, fail to associate a character to a single, most relevant, occupation.

Pantheon 2.0 follows a similar hierarchical occupation classification that Pantheon 1.0—e.g. Physics and Biology are branches of Natural Science, just like Natural Science is a branch of Science. Pantheon 1.0 has 88 different occupations at the lowest level of aggregation. Pantheon 2.0 adds 13 new occupations: youtuber, including people such as PewDiePie who are YouTube celebrities, occultist, including people such as Nostradamus who are said to have paranormal powers, inspiration, including people such as Vlad the Impaler who are famous for serving as inspiration for fictional characters, and the 10 new sports categories, badminton player, rugby player, handball player, bullfighter, volleyball player, pc gamer, poker player, go player, fencer, and table tennis player. A full list of all the occupations can be found in Table 8.

We use a SVM classifier, trained on the Pantheon 1.0 dataset, to classify the biographies of Pantheon 2.0. Since 13 new occupations were added in Pantheon 2.0, we complement Pantheon 1.0 with manually classified characters for each new occupation in order to build the training set.

The features we select for the classifier are drawn from Wikidata and Wikipedia, and are meant to characterize the character's main field of contribution. We select the following features:

- Infobox type: The type of the infobox templates used in the wikipage associated with the character. We filter out all the infoboxes that do not correspond to a biography.
- Wikidata occupations: For each character we collect all the values from the property "occupation" from Wikidata. We manually created a controlled vocabulary of 350 occupations—e.g. design engineer is mapped to engineer, marine biologist to biologist, etc.
- Extract words: We get the top 5 most frequent words from the character's extract, selected from a manually curated list of 750 words that are meant to capture the character's work. For example: backstroke, summit, reign, contribution, youtube, football, testament, etc.

When testing the classifier by setting aside 10% of the data, we get $\sim 93\%$ success rate. The success rate is slightly higher in the second level of aggregation ($\sim 95\%$), which is the level we use to obtain the results reported in this work. Pantheon

2.0 classifies characters into 8 *domains*, 27 *industries*, and 101 *occupations*. For our analysis, we aggregate occupations into categories distinguishing mainly between politicians and religious figures, and between arts and performing arts. The *arts* domain is split into two groups *performing arts* (including *dance*, and *film and theater* industries, plus all occupations from the *music* industry except for *composer*) and *arts* (including *design* and *fine arts* industries and the *composer* occupation). The *religion* industry is grouped by itself, and all the other industries under the *institutions* domain are grouped together under *government*. The *team sports* industry is considered under *sports*. The *science and technology*, and *humanities* industries remain unchanged. Finally, *individual sports*, along with the domains *business and law*, *exploration*, and *public figure* are grouped together as *other*. Table 8 shows a summary of the aforementioned aggregation. We must note that we are not loosing meaningful information by creating the category *other*. The three largest occupations aggregated under *other*—*tennis player*, *social activist*, and *racecar driver*—are very small—with 161, 114, and 104 individuals respectively. Therefore, any change in the category *other* will also be captured by other categories. For example, there is an observed increase in the number of *tennis players* in the second half of the 1900s due to the adoption of television, but this change is already captured by the *sports* category.

A.2 Population and technology adoption

Population data comes from two sources. At the global level we use data from the historical world population estimates of the US Census Bureau (2), which reports an aggregated dataset of world population estimates starting from the year 10,000 BC. At the city level we use a dataset on population of urban settlements from 3700 BC to AD 2000 (3). We matched the cities present in GeoNames with the ones used in (3) based on their name, within a 30km radius. We interpolate the missing years for both population datasets using linear splines.

Data on technology adoption comes from two sources. For printing, we use the *Incunabula Short Title Catalogue* (4), a dataset comprising all books printed between 1450 and 1500. For radio and for television we use the Historical Cross-Country Technology Adoption (HCCTA) dataset (5), a dataset collected to analyze the adoption patterns of some of the major technologies introduced in the past 250 years. In particular we use the variables *Radios*, and *Televisions*. The HCCTA dataset also has historical GDP and population information.

A.3 Changepoint analysis

The claim in the main text that printing coincides with a sharp increase in the per-capita number of memorable people born each year is supported by a technique used in time series analysis to detect abrupt changes in the mean of the series. The *changepoint* estimation technique (6) estimates the position and number of changepoints in a time series by assuming that the time series can be modeled by a distribution with a fixed mean. The changepoints in a time series are the points that require updating the mean of the distribution used to model the data. To find the changepoints, the technique minimizes a test statistic that depends on the number and position of the changepoints. All changepoint analyses were performed using the *changepoint* package available for R (6).

A.4 Distance to Mainz as an IV

As pointed out in the main text, we are by no means interested in explaining the spread of the printing press. Instead, we are interested in the effects of the spread of printing. The problem is that we believe that the relation we are trying to establish is highly endogenous. To solve this endogeneity, we use the distance to Mainz as an *instrumental variable* (7). An instrumental variable is an external variable that affects the explanatory variable, but does not affect the outcome variable directly. The distance to Mainz is a good instrumental variable because it affect the adoption of printing, but does not directly affect a city's ability to produce famous scientist and artists. This condition is often referred to as the *exclusion restriction*.

As is usual when using instrumental variable analysis, our main result depends on the validity of the exclusion restriction. Luckily, there are not many mechanisms, other than the spread of the printing press, by which the distance to Mainz can correlate with the number of scientists and artists born in a city. Yet, one might still think of other technologies that might have "radiated" from Mainz, since Mainz was a relatively important city at the time. For example, the presence of universities or churches can be argued to function as infrastructure for the arts and sciences, and they might correlate with the distance to Mainz. To rule out this possibility, we analyze the correlation between the distance to Mainz and the number of scientists and artists born in a city, before and after the introduction of the printing press. In other words, we focus on the *reduced form* of the two stage least squares regressions presented in Table 1 of the main text. Table 1 shows that the distance to Mainz correlates with the number of scientists and artists only after the introduction of printing, and it does not correlate with the number of political leaders. The

before period is calculated between 1250 and 1350 because we are dealing with birth years, and hence we need a period of people that had a chance to complete their careers before printing was introduced. Table **??** shows the reduced forms for the regressions presented in Table 2 from the main text for before and after printing. Here we use the period between 1200 and 1400 as the before period, because we are only focusing on the first scientist and artist born in the city, and because the period after printing presented in the main text spans also 200 years, from 1400 to 1600. Together, both tables support the validity of the exclusion restriction by providing evidence that the correlation between the distance to Mainz and the number of scientists and artists is in fact due to the spread of printing and not to other forms of infrastructure, which were already present before the invention of printing.

Table 1. Correlation between distance to Mainz and number of artists and scientists born in a city before and after the invention of printing. Before period is between 1200 and 1350, and after period between 1400 and 1550. The correlation between distance to Mainz and the number of scientists and artists only appears after the introduction of the printing press.

	Dependent variable:						
	printer	number of scientists		number of artists		number of political leaders	
	First Stage	*before printing*	*after printing*	*before printing*	*after printing*	*before printing*	*after printing*
	(1)	(2)	(3)	(4)	(5)	(6)	(7)
distance to Mainz	−0.022***	0.00002	−0.003***	−0.001	−0.006***	−0.002	−0.002
	(0.003)	(0.0002)	(0.001)	(0.001)	(0.001)	(0.002)	(0.002)
constant	0.186***	0.0002	0.019***	0.005	0.047***	0.021*	0.030**
	(0.018)	(0.001)	(0.004)	(0.004)	(0.010)	(0.010)	(0.011)
Observations	5,335	5,335	5,335	5,335	5,335	5,335	5,335
R^2	0.012	0.00000	0.004	0.0002	0.003	0.0002	0.0003
Adjusted R^2	0.012	−0.0002	0.004	0.00004	0.003	0.00003	0.0002
Residual SE (df = 5333)	0.187	0.013	0.036	0.038	0.098	0.104	0.115
F Statistic (df = 1; 5333)	66.569***	0.007	22.944***	1.217	16.273***	1.145	1.864
Note:					*p<0.05; **p<0.01; ***p<0.001		

Table 2. Correlation between the distance to Mainz and the year of the first scientist and artist, before and after the invention of printing. Before period is between 1150 and 1350, and after period between 1400 and 1600. The correlation between the distance to Mainz and the year of the first scientist and the first artist only appears ater the introduction of the printing press.

	Dependent variable:						
	year of first printer	year of first scientist		year of first artist		year of first political leader	
	First Stage	*before printing*	*after printing*	*before printing*	*after printing*	*before printing*	*after printing*
	(1)	(2)	(3)	(4)	(5)	(6)	(7)
distance to Mainz	0.010**	0.034	0.112**	0.037	0.105*	−0.035	−0.021
	(0.003)	(0.058)	(0.042)	(0.055)	(0.049)	(0.061)	(0.053)
population	−3.280*	−32.382	−44.511*	−69.048*	−63.321*	−126.139***	−61.910*
	(1.644)	(26.221)	(20.550)	(28.594)	(26.396)	(32.678)	(29.220)
constant	55.853***	526.733*	628.257**	810.620**	762.471**	1,332.769***	797.214**
	(16.207)	(256.972)	(203.430)	(279.572)	(259.817)	(318.331)	(287.126)
Observations	82	66	73	64	70	70	77
R^2	0.132	0.025	0.114	0.088	0.096	0.217	0.082
Adjusted R^2	0.111	−0.006	0.089	0.058	0.069	0.193	0.057
Residual SE	10.611	170.876	130.430	180.671 153.785	216.842	184.058	
Residual SE df	79	63	70	61	67	67	74
F Statistic	6.032**	0.803	4.512*	2.926	3.565*	9.268***	3.291*
F Statistic df	2; 79	2; 63	2; 70	2; 61	2; 67	2; 67	2; 74
Note:							*p<0.05; **p<0.01; ***p<0.001

B Supplementary results

B.1 Printing and the rise of the arts and sciences

In this section, we will explore three different empirical specifications that allow us to establish the relation between early adoption of printing and the production of memorable scientists and artists.

First, we use the distance to Mainz as an instrumental variable for a dummy variable for whether a city adopted printing between 1450 and 1500, D_{printer}. As dependent variables we use the number of people born in each city between 1400 and 1550, for each occupation. Tables 3 show the result of this analysis for all occupations.

Second, we use the distance to Mainz as an instrumental variable for the year of the first printed book in each city. As dependent variables we use the year of the first person born in each city, after 1400, for each occupation. Tables 4 show the results for all occupations. Here we consider only cities that adopted printing and for which we have population data.

Finally, we use the distance to Mainz as an instrumental variable for the number of printed books between 1450 and 1500. As dependent variables we use the number of people born in each city between 1450 and 1550 (right after the invention of printing in Europe) for each occupation. Tables 5 show the results of this analysis for all occupations. We consider only cities that adopted printing (i.e. $n_{\text{books}} > 0$) and for which we have population data.

B.2 Radio and television and the rise of entertainment

We regress the number of people born in each country in each year (between 1820 and 1998), for each category, against the number of televisions and the number of radios, controlling for GDP and population. As mentioned before, gata on GDP, population, number of radios, and number of televisions comes from the HCCTA dataset (5). Tables 6 and 7 show the results for all occupations. The differences between the model with and without the number of radios and televisions are significant for performing arts, sports players, political leaders, and humanities (p-values of $\sim$ 1e-16, $\sim$ 1e-16, $\sim$ 1e-06, and $\sim$ 1e-03, respectively), and are not significant for scientists, artists, and religious leaders (p-values of 0.1865, 0.0308, and 0.1938, respectively). We note that the category humanities includes journalists (see Table 8).

Table 3. Instrumental variable analysis of the effect of printing on the number of memorable characters. Here, we use the distance to Mainz as an instrument for a dummy variable for adopting printing between 1450 and 1500, to estimate the effect of printing in the number of scientists, artists, political leaders, humanities, religious leaders, and all people in the dataset born between 1450 and 1550. All dependent variables are in log-scale.

	Dependent variable:							
	printing before 1500		number of scientists		number of artists		number of political leaders	
	probit	*First Stage*	*OLS*	*IV*	*OLS*	*IV*	*OLS*	*IV*
	(1)	(2)	(3)	(4)	(5)	(6)	(7)	(8)
distance to Mainz	-0.273^{***}	-0.022^{***}						
	(0.033)	(0.003)						
printing before 1500			0.089^{***}	0.222^{***}	0.202^{***}	0.302^{***}	0.188^{***}	0.130
			(0.004)	(0.043)	(0.007)	(0.067)	(0.009)	(0.081)
constant	-0.014	0.186^{***}	0.002^{**}	-0.003	0.003^{**}	-0.0002	0.010^{***}	0.012^{***}
	(0.212)	(0.018)	(0.001)	(0.002)	(0.001)	(0.003)	(0.002)	(0.003)
Observations	5,335	5,335	5,335	5,335	5,335	5,335	5,335	5,335
Log Likelihood	-804.19							
AIC	1,612							
Residual SE (df = 5333)		0.187	0.061	0.066	0.100	0.102	0.124	0.124
R^2		0.012	0.070		0.125		0.075	
Adjusted R^2		0.012	0.070		0.125		0.075	
F Statistic (df = 1; 5333)		66.57^{***}	399.43^{***}		762.57^{***}		435.08^{***}	
Note:						*p<0.1; **p<0.05; ***p<0.01		

	Dependent variable:					
	number of humanities		number of religious leaders		number of people	
	OLS	*IV*	*OLS*	*IV*	*OLS*	*IV*
	(9)	(10)	(11)	(12)	(13)	(14)
distance to Mainz						
printing before 1500	0.103^{***}	0.058	0.067^{***}	0.057	0.505^{***}	0.663^{***}
	(0.006)	(0.050)	(0.005)	(0.042)	(0.014)	(0.130)
constant	0.004^{***}	0.005^{**}	0.002^{***}	0.003	0.024^{***}	0.018^{***}
	(0.001)	(0.002)	(0.001)	(0.002)	(0.003)	(0.005)
Observations	5,335	5,335	5,335	5,335	5,335	5,335
Residual SE (df = 5333)	0.075	0.076	0.065	0.065	0.196	0.199
R^2	0.062		0.036		0.189	
Adjusted R^2	0.062		0.036		0.189	
F Statistic (df = 1; 5333)	353.06^{***}		201.79^{***}		$1,243^{***}$	
Note:				*p<0.1; **p<0.05; ***p<0.01		

Table 4. Instrumental variable analysis of the effect of printing on cities that adopted printing. Here, we use the distance to Mainz as an instrument for the year of the first printing press in each city, to estimate the effect of printing in the birth of scientists, artists, political leaders, performing artists, humanities, religious leaders, and sports players. The two stage least squares estimates show that cities that adopted printing earlier were the birth place of scientists and artists earlier than late adopters, but not of political leaders, performing artists, humanities, religious leaders, or sports players. Population variable corresponds to the average city population between 1400 and 1600.

	Dependent variable:						
	year of first printer	year of first scientist		year of first artist		year of first political leader	
	First Stage	*OLS*	*IV*	*OLS*	*IV*	*OLS*	*IV*
	(1)	(2)	(3)	(4)	(5)	(6)	(7)
distance to Mainz	0.010^{***}						
	(0.003)						
year of first printer		4.870^{***}	12.900^{**}	6.552^{***}	12.956^{**}	3.036^{*}	-1.955
		(1.425)	(5.655)	(1.740)	(6.194)	(1.809)	(4.988)
population	-3.280^{**}	-18.507	-5.089	-37.476^{*}	-38.083	-64.311^{**}	-68.651^{**}
	(1.644)	(19.035)	(24.651)	(21.950)	(24.072)	(26.015)	(27.613)
constant	55.853^{***}	294.220	-69.230	383.895	215.241	719.812^{**}	908.366^{***}
	(16.207)	(203.821)	(346.180)	(231.540)	(297.534)	(275.779)	(337.974)
Observations	82	73	73	70	70	77	77
Residual SE	10.611	126.760	152.822	144.475	158.400	180.853	189.924
Residual SE df	79	70	70	67	67	74	74
R^2	0.132	0.163		0.202		0.113	
Adj. R^2	0.111	0.139		0.178		0.089	
F Statistic	6.032^{***}	6.833^{***}		8.496^{***}		4.732^{**}	
F Statistic df	2; 79	2; 70		2; 67		2; 74	
Note:						*p<0.1; **p<0.05; ***p<0.01	

	Dependent variable:							
	year of first performing artist		year of first humanities		year of first religious leader		year of first sports player	
	OLS	*IV*	*OLS*	*IV*	*OLS*	*IV*	*OLS*	*IV*
	(8)	(9)	(10)	(11)	(12)	(13)	(14)	(15)
distance to Mainz								
year of first printer	2.770^{***}	3.397	4.327^{***}	2.845	0.880	1.790	0.891^{***}	1.273^{*}
	(0.910)	(2.675)	(1.510)	(4.076)	(2.770)	(7.350)	(0.261)	(0.678)
population	-30.36^{**}	-29.45^{**}	-59.85^{***}	-61.51^{***}	-82.86^{**}	-82.65^{**}	-8.55^{**}	-8.27^{**}
	(13.055)	(13.599)	(21.637)	(22.189)	(31.069)	(31.144)	(3.883)	(3.968)
constant	647.2^{***}	619.7^{***}	705.3^{***}	765.5^{***}	$1{,}073^{***}$	$1{,}048^{***}$	556.15^{***}	542.2^{***}
	(139.3)	(178.1)	(230.4)	(278.3)	(331.0)	(381.4)	(41.14)	(47.58)
Observations	71	71	77	77	48	48	74	74
Residual SE	87.755	88.061	151.417	152.399	185.473	185.695	26.127	26.519
Residual SE df	68	68	74	74	45	45	71	71
R^2	0.193		0.189		0.139		0.196	
Adj. R^2	0.169		0.167		0.100		0.173	
F Statistic	8.136^{***}		8.606^{***}		3.625^{**}		8.650^{***}	
F Statistic df	2; 68		2; 74		2; 45		2; 71	
Note:							*p<0.1; **p<0.05; ***p<0.01	

Table 5. Instrumental variable analysis of the effect of the number of printed books. Here, we use the distance to Mainz as an instrument for the number of books printed in each city between 1450 and 1500, restricted to cities that adopted printing (i.e. $n_{\text{books}} > 0$). We use a two stage least squares regression to estimate the effect of printing in the number of scientists (models 2 and 3), artists (models 4 and 5), political leaders (models 6 and 7), humanities (models 8 and 9), religious leaders (models 10 and 11), and all people in the dataset (models 12 and 13), born between 1450 and 1550. All dependent variables are in log-scale.

	Dependent variable:						
	number of books	number of scientists		number of artists		number of political leaders	
	First Stage	*OLS*	*IV*	*OLS*	*IV*	*OLS*	*IV*
	(1)	(2)	(3)	(4)	(5)	(6)	(7)
distance to Mainz	-0.948^{***}						
	(0.314)						
n. of books		0.090^{***}	0.184^{***}	0.149^{***}	0.235^{**}	0.076^{*}	-0.166
		(0.019)	(0.068)	(0.033)	(0.105)	(0.041)	(0.152)
population	1.082^{***}	0.105^{*}	0.029	0.276^{***}	0.207	0.331^{***}	0.527^{***}
	(0.303)	(0.055)	(0.081)	(0.092)	(0.125)	(0.115)	(0.181)
constant	-1.735	-1.191^{**}	-0.730	-2.893^{***}	-2.471^{**}	-2.959^{**}	-4.147^{***}
	(3.142)	(0.545)	(0.698)	(0.916)	(1.074)	(1.148)	(1.552)
Observations	81	81	81	81	81	81	81
Residual SE (df = 78)	1.908	0.345	0.394	0.581	0.607	0.728	0.877
R^2	0.178	0.297		0.346		0.171	
Adjusted R^2	0.157	0.279		0.329		0.150	
F Statistic (df = 2; 78)	8.458^{***}	16.442^{***}		20.637^{***}		8.050^{***}	
Note:							*p<0.1; **p<0.05; ***p<0.01

	Dependent variable:					
	number of humanities		number of religious leaders		number of people	
	OLS	*IV*	*OLS*	*IV*	*OLS*	*IV*
	(8)	(9)	(10)	(11)	(12)	(13)
distance to Mainz						
n. of books	0.084^{***}	0.023	0.065^{***}	0.017	0.214^{***}	0.102
	(0.023)	(0.075)	(0.023)	(0.073)	(0.045)	(0.144)
population	0.267^{***}	0.317^{***}	0.189^{***}	0.228^{**}	0.502^{***}	0.592^{***}
	(0.066)	(0.089)	(0.065)	(0.087)	(0.126)	(0.171)
constant	-2.707^{***}	-3.008^{***}	-1.941^{***}	-2.176^{***}	-4.602^{***}	-5.152^{***}
	(0.655)	(0.767)	(0.643)	(0.742)	(1.260)	(1.470)
Observations	81	81	81	81	81	81
Residual SE (df = 78)	0.415	0.433	0.408	0.419	0.799	0.831
R^2	0.347		0.229		0.408	
Adjusted R^2	0.330		0.209		0.393	
F Statistic (df = 2; 78)	20.738^{***}		11.599^{***}		26.919^{***}	
Note:						*p<0.1; **p<0.05; ***p<0.01

Table 6. Impact of the adoption of radio and television on the number of people born, for each category, between 1820 and 1998. All dependent variables are in log-scale, and are calculated yearly. The differences between models (2) and (3), and (5) and (6) are significant, while the difference between models (8) and (9) are not.

	Dependent variable:								
	number of performing artists			number of sports players			number of scientists		
	(1)	(2)	(3)	(4)	(5)	(6)	(7)	(8)	(9)
n. of radios	0.037^{***}		0.031^{***}	0.027^{***}		0.029^{***}	0.003		-0.003
	(0.005)		(0.005)	(0.004)		(0.004)	(0.004)		(0.004)
n. of tvs	0.079^{***}		0.072^{***}	0.040^{***}		0.042^{***}	-0.001		-0.007
	(0.009)		(0.009)	(0.006)		(0.006)	(0.006)		(0.006)
GDP		0.477^{***}	0.312^{***}		0.091^{*}	-0.033		0.218^{***}	0.235^{***}
		(0.069)	(0.067)		(0.050)	(0.050)		(0.043)	(0.044)
population		-0.283^{***}	-0.158^{**}		-0.135^{**}	-0.041		-0.044	-0.056
		(0.070)	(0.068)		(0.053)	(0.052)		(0.044)	(0.044)
constant	-0.058	-0.268	-0.323	-0.107	-0.283	-0.385	-0.027	-0.514	-0.580
	(0.074)	(0.308)	(0.303)	(0.107)	(0.368)	(0.369)	(0.097)	(0.366)	(0.362)
Observations	3,011	3,011	3,011	3,011	3,011	3,011	3,011	3,011	3,011
R^2	0.342	0.353	0.354	0.498	0.494	0.499	0.519	0.523	0.525
Adj. R^2	0.301	0.313	0.314	0.467	0.463	0.468	0.489	0.494	0.495
Residual SE	0.331	0.328	0.328	0.420	0.421	0.419	0.386	0.384	0.384
Residual SE df	2835	2835	2833	2835	2835	2833	2835	2835	2833
F Statistic	8.408^{***}	8.834^{***}	8.789^{***}	16.061^{***}	15.836^{***}	15.955^{***}	17.464^{***}	17.760^{***}	17.692^{***}
F Statistic df	175; 2835	175; 2835	177; 2833	175; 2835	175; 2835	177; 2833	175; 2835	175; 2835	177; 2833

Note: *p<0.1; **p<0.05; ***p<0.01

Table 7. Impact of the adoption of radio and television on the number of people born, for each category, between 1820 and 1998. All dependent variables are in log-scale, and are calculated yearly. The differences between models (14) and (15), and (17) and (18) are significant, while the difference between models (11) and (12), and (20) and (21) are not.

	Dependent variable:					
		number of artists			number of political leaders	
	(10)	(11)	(12)	(13)	(14)	(15)
n. of radios	0.007**		0.003	0.005		0.003
	(0.003)		(0.003)	(0.004)		(0.004)
n. of tvs	0.014**		0.010*	0.029***		0.027***
	(0.006)		(0.005)	(0.007)		(0.007)
GDP		0.204***	0.184***		0.114**	0.072
		(0.040)	(0.040)		(0.052)	(0.052)
population		−0.096**	−0.081**		−0.050	−0.019
		(0.040)	(0.040)		(0.051)	(0.052)
constant	−0.058	−0.268	−0.323	−0.107	−0.283	−0.385
	(0.074)	(0.308)	(0.303)	(0.107)	(0.368)	(0.369)
Observations	3,011	3,011	3,011	3,011	3,011	3,011
R^2	0.342	0.353	0.354	0.498	0.494	0.499
Adj. R^2	0.301	0.313	0.314	0.467	0.463	0.468
Residual SE	0.331	0.328	0.328	0.420	0.421	0.419
Residual SE df	2835	2835	2833	2835	2835	2833
F Statistic	8.408***	8.834***	8.789***	16.061***	15.836***	15.955***
F Statistic df	175; 2835	175; 2835	177; 2833	175; 2835	175; 2835	177; 2833
Note:						*p<0.1; **p<0.05; ***p<0.01

	Dependent variable:					
		number of humanities			number of religious leaders	
	(16)	(17)	(18)	(19)	(20)	(21)
n. of radios	0.006		0.002	0.003		0.003
	(0.004)		(0.004)	(0.003)		(0.003)
n. of tvs	0.020***		0.017***	−0.001		−0.001
	(0.006)		(0.006)	(0.003)		(0.003)
GDP		0.173***	0.146***		0.010	0.004
		(0.045)	(0.045)		(0.021)	(0.022)
population		−0.059	−0.039		−0.007	−0.002
		(0.045)	(0.045)		(0.021)	(0.021)
constant	−0.027	−0.514	−0.580	−0.051**	−0.030	−0.056
	(0.097)	(0.366)	(0.362)	(0.020)	(0.147)	(0.148)
Observations	3,011	3,011	3,011	3,011	3,011	3,011
R^2	0.519	0.523	0.525	0.181	0.180	0.181
Adj. R^2	0.489	0.494	0.495	0.131	0.130	0.130
Residual SE	0.386	0.384	0.384	0.194	0.195	0.194
Residual SE df	2835	2835	2833	2835	2835	2833
F Statistic	17.464***	17.760***	17.692***	3.582***	3.559***	3.539***
F Statistic df	175; 2835	175; 2835	177; 2833	175; 2835	175; 2835	177; 2833
Note:						*p<0.1; **p<0.05; ***p<0.01

C Table of occupations

Table 8. Description of Pantheon 2.0 categories and the aggregation used in our analysis.

Occupation	Category	Number of people
COMIC ARTIST	ARTIST	103
GAME DESIGNER	ARTIST	25
ARTIST	ARTIST	51
PHOTOGRAPHER	ARTIST	57
DESIGNER	ARTIST	45
PAINTER	ARTIST	917
SCULPTOR	ARTIST	105
FASHION DESIGNER	ARTIST	35
ARCHITECT	ARTIST	273
COMPOSER	ARTIST	757
CRITIC	HUMANITIES	5
JOURNALIST	HUMANITIES	83
LINGUIST	HUMANITIES	94
PHILOSOPHER	HUMANITIES	638
WRITER	HUMANITIES	3317
HISTORIAN	HUMANITIES	171
GAMER	OTHER	1
SOCIAL ACTIVIST	OTHER	306
EXTREMIST	OTHER	129
YOUTUBER	OTHER	9
BADMINTON PLAYER	OTHER	21
PRODUCER	OTHER	56
MAGICIAN	OTHER	10
POKER PLAYER	OTHER	7
ATHLETE	OTHER	1180
LAWYER	OTHER	29
MOUNTAINEER	OTHER	23
MARTIAL ARTS	OTHER	51
PORNOGRAPHIC ACTOR	OTHER	200
TABLE TENNIS PLAYER	OTHER	28
PIRATE	OTHER	18
COMPANION	OTHER	497
GYMNAST	OTHER	72
EXPLORER	OTHER	297
CELEBRITY	OTHER	137
CHESS PLAYER	OTHER	173
BULLFIGHTER	OTHER	1
INSPIRATION	OTHER	6
OCCULTIST	OTHER	5
BOXER	OTHER	92
MAFIOSO	OTHER	38
HANDBALL PLAYER	OTHER	55
GOLFER	OTHER	34
CHEF	OTHER	4
MODEL	OTHER	133

ASTRONAUT	OTHER	400
SWIMMER	OTHER	171
WRESTLER	OTHER	285
TENNIS PLAYER	OTHER	804
FENCER	OTHER	23
SKIER	OTHER	225
GO PLAYER	OTHER	2
RACING DRIVER	OTHER	666
CYCLIST	OTHER	527
SKATER	OTHER	130
SNOOKER	OTHER	22
PRESENTER	OTHER	70
BUSINESSPERSON	OTHER	326
FILM DIRECTOR	PERFORMING ARTISTS	882
DANCER	PERFORMING ARTISTS	48
MUSICIAN	PERFORMING ARTISTS	1621
COMEDIAN	PERFORMING ARTISTS	8
ACTOR	PERFORMING ARTISTS	5450
SINGER	PERFORMING ARTISTS	2175
CONDUCTOR	PERFORMING ARTISTS	92
POLITICIAN	POLITICAL LEADERS	8438
PILOT	POLITICAL LEADERS	24
MILITARY PERSONNEL	POLITICAL LEADERS	806
NOBLEMAN	POLITICAL LEADERS	287
JUDGE	POLITICAL LEADERS	27
DIPLOMAT	POLITICAL LEADERS	42
PUBLIC WORKER	POLITICAL LEADERS	13
RELIGIOUS FIGURE	RELIGIOUS LEADERS	1307
PHYSICIST	SCIENTIST	518
INVENTOR	SCIENTIST	201
CHEMIST	SCIENTIST	393
BIOLOGIST	SCIENTIST	462
ECONOMIST	SCIENTIST	218
ARCHAEOLOGIST	SCIENTIST	52
GEOGRAPHER	SCIENTIST	44
ASTRONOMER	SCIENTIST	357
MATHEMATICIAN	SCIENTIST	539
PSYCHOLOGIST	SCIENTIST	134
STATISTICIAN	SCIENTIST	9
COMPUTER SCIENTIST	SCIENTIST	141
GEOLOGIST	SCIENTIST	36
ENGINEER	SCIENTIST	226
ANTHROPOLOGIST	SCIENTIST	30
PHYSICIAN	SCIENTIST	320
POLITICAL SCIENTIST	SCIENTIST	23
SOCIOLOGIST	SCIENTIST	37
VOLLEYBALL PLAYER	SPORTS PLAYERS	47
HOCKEY PLAYER	SPORTS PLAYERS	99
COACH	SPORTS PLAYERS	115
BASEBALL PLAYER	SPORTS PLAYERS	25
SOCCER PLAYER	SPORTS PLAYERS	6482
REFEREE	SPORTS PLAYERS	65

AMERICAN FOOTBALL PLAYER	SPORTS PLAYERS	24
CRICKETER	SPORTS PLAYERS	21
BASKETBALL PLAYER	SPORTS PLAYERS	670
RUGBY PLAYER	SPORTS PLAYERS	8

References

1. Wick M, Vatant B. The geonames geographical database. Available from World Wide Web: http://geonames org. 2012;.
2. Bureau USC. World Population; 2015. Available from: http://www.census.gov/population/international/data/worldpop/table_population.php.
3. Reba M, Reitsma F, Seto KC. Spatializing 6,000 years of global urbanization from 3700 BC to AD 2000. Scientific data. 2016;3:160034.
4. ISTC. Incunabula Short Title Catalogue. British Library; 1998.
5. Comin D, Hobijn B. Cross-country technology adoption: making the theories face the facts. Journal of monetary Economics. 2004;51(1):39–83.
6. Killick R, Eckley I. Changepoint: An R package for changepoint analysis. Journal of Statistical Software. 2014;58(3):1–19.
7. Angrist JD, Pischke JS. Instrumental Variables in Action: Sometimes You Get What You Need. In: Mostly harmless econometrics: An empiricist's companion. Princeton university press; 2008.
